# An external validation of models to predict the onset of chronic kidney disease using population-based electronic health records from Salford, UK

**DOI:** 10.1186/s12916-016-0650-2

**Published:** 2016-07-12

**Authors:** Paolo Fraccaro, Sabine van der Veer, Benjamin Brown, Mattia Prosperi, Donal O’Donoghue, Gary S. Collins, Iain Buchan, Niels Peek

**Affiliations:** NIHR Greater Manchester Primary Care Patient Safety Translational Research Centre, Institute of Population Health, The University of Manchester, Manchester, UK; Health eResearch Centre, Farr Institute for Health Informatics Research, Manchester, UK; Centre for Health Informatics, Institute of Population Health, The University of Manchester, Vaughan House, Portsmouth St, Manchester, M13 9GB UK; Department of Epidemiology, University of Florida, Gainesville, FL USA; Renal Clinic, Salford Royal NHS Trust, Salford, UK; Centre for Statistics in Medicine, Nuffield Department of Orthopaedics, Rheumatology & Musculoskeletal Sciences, University of Oxford, Oxford, UK

**Keywords:** Chronic kidney disease, Clinical prediction models, eGFR, Decision support, Electronic health records, Model validation, Model calibration

## Abstract

**Background:**

Chronic kidney disease (CKD) is a major and increasing constituent of disease burdens worldwide. Early identification of patients at increased risk of developing CKD can guide interventions to slow disease progression, initiate timely referral to appropriate kidney care services, and support targeting of care resources. Risk prediction models can extend laboratory-based CKD screening to earlier stages of disease; however, to date, only a few of them have been externally validated or directly compared outside development populations. Our objective was to validate published CKD prediction models applicable in primary care.

**Methods:**

We synthesised two recent systematic reviews of CKD risk prediction models and externally validated selected models for a 5-year horizon of disease onset. We used linked, anonymised, structured (coded) primary and secondary care data from patients resident in Salford (population ~234 k), UK. All adult patients with at least one record in 2009 were followed-up until the end of 2014, death, or CKD onset (*n* = 178,399). CKD onset was defined as repeated impaired eGFR measures over a period of at least 3 months, or physician diagnosis of CKD Stage 3–5. For each model, we assessed discrimination, calibration, and decision curve analysis.

**Results:**

Seven relevant CKD risk prediction models were identified. Five models also had an associated simplified scoring system. All models discriminated well between patients developing CKD or not, with c-statistics around 0.90. Most of the models were poorly calibrated to our population, substantially over-predicting risk. The two models that did not require recalibration were also the ones that had the best performance in the decision curve analysis.

**Conclusions:**

Included CKD prediction models showed good discriminative ability but over-predicted the actual 5-year CKD risk in English primary care patients. QKidney, the only UK-developed model, outperformed the others. Clinical prediction models should be (re)calibrated for their intended uses.

**Electronic supplementary material:**

The online version of this article (doi:10.1186/s12916-016-0650-2) contains supplementary material, which is available to authorized users.

## Background

Chronic kidney disease (CKD) presents a substantial burden of disease worldwide [1–4], with an increasing number of people being diagnosed [5, 6]. A 2010 study of 2.8 UK adults reported a 5.9 % prevalence of stage 3–5 CKD [7]. In the UK, costs related to CKD care in 2009–2010 were estimated around £1.45 billion (1.3 % of the National Health Service (NHS) budget) [8] – costs that are set to rise steeply [6, 8].

Early detection of CKD, and identification of patients at increased risk of developing CKD, can improve care by guiding preventive measures to slow disease progression, initiating timely referral to nephrology care, and supporting better allocation of resources [9]. Yet, despite worldwide efforts to improve detection [10], CKD often remains undiagnosed in its early stages [5]. Currently, most CKD clinical surveillance relies on estimated Glomerular Filtration Rate (eGFR) from serum creatinine testing [10]. In the UK, national clinical practice guidelines recommend systematic monitoring, in the primary care setting, of eGFR in patients with CKD risk factors (i.e. diabetes, hypertension, cardiovascular diseases, or use of particular medications) [11]. In addition, eGFR has been calculated routinely in UK NHS laboratories since 2006, where at least age, sex and creatinine variables are available – so CKD may be picked up in a variety of clinical contexts. Nevertheless, the value of universal clinical/opportunistic screening for CKD remains unclear [12].

Risk prediction models can extend the clinical screening toolkit from measured to predicted disease, affording more timely intervention, for example, to reduce risk factors [13]. Several models have been developed to predict CKD onset, but most have not been validated outside the setting in which they were developed [14, 15]. Therefore, the portability of these models to other populations, risk environments and healthcare settings has yet to be demonstrated. Furthermore, comprehensive head-to-head comparisons of these purportedly alternative models are lacking in the literature [14–16]. Only one comparison of two CKD prediction models in a small cohort was published to date [17].

The aim of this study was to externally validate and compare the performance of previously published models for predicting 5-year CKD risk using routine healthcare records from a UK population with well-studied, high quality electronic health records.

## Methods

### Reporting

The reporting of this external validation study follows the TRIPOD statement [18, 19], which is a set of recommendations for the reporting of studies describing the development, validation, or updating of prediction models [18, 19].

### Literature review

Two recent systematic reviews identified prediction models on CKD onset and CKD progression [14, 15]. From these reviews, we selected models predicting CKD onset that could be used in primary care. Models were excluded if (1) they were developed for a specific subpopulation (e.g. HIV patients [20]); (2) the covariate coefficients and regression formula were not reported in the original study; or (3) they had more than one predictor not routinely collected in UK primary care (more than one predictor for which we had > 70 % missing data in our dataset).

Where available, we included simplified scoring systems accompanying the included prediction models. Such systems typically produce an integer score for each patient, where higher scores represent higher predicted risk but there is no relationship with absolute risk.

### Validation cohort

#### Outcome

The outcome of interest was onset of CKD within 5 years. Existing models employ various definitions of CKD [14, 15]. For our study, we followed international guidelines [21] and considered a recent study [7] reporting UK CKD prevalence based on primary care records. We defined CKD as (1) the presence of at least two consecutive eGFR values below 60 mL/min/1.73 m^2^, as calculated with the Modification of Diet in Renal Disease (MDRD) formula [22], over a period of 3 months or longer; or (2) the presence of a CKD Stage 3–5 diagnostic code.

We were unable to incorporate albumin-creatinine ratio (ACR, a predictor of kidney damage [23] noted in international guidelines [21]) because ACR data are available only for selected groups of patients at risk of CKD, such as those on diabetes care pathways.

#### Data source

We used linked, anonymised data from the Salford Integrated Record (SIR) up to the end of 2014. SIR is an electronic health record (EHR) that has been overlain on primary and secondary care clinical information systems for over 10 years in the city of Salford (population 234 k) – an early-adopter site of healthcare IT in the UK. SIR includes patient records submitted by all 53 primary care providers and the one secondary care provider for this population, stored as Read codes versions 2 and 3 [24]. The data cover all primary care, some of secondary care – focused on long-term conditions management – and all results from biochemical testing across primary and secondary care.

#### Study population

Salford is a relatively deprived population with a high burden of disease, where the EHR data have been used extensively to study the population’s health and care. Like all English localities, Salford’s primary care is measured and remunerated under the Quality and Outcomes Framework, including counts of the mean number of conditions per registered patient, where Salford falls in 61st centile [25].

We included all adults (aged 18 years or older) registered with a Salford practice with at least one record in SIR between April 1, 2009, and March 31, 2010 – the financial year. We looked at the financial rather than calendar year to take account of the Quality and Outcomes Framework, which might have influenced the quality of data recorded by GPs [26, 27]. For all retrieved patients the entry date was the date of the first record in the financial year 2009. Included patients were followed until December 31, 2014, or censored when they moved outside of Salford or died.

We excluded patients with CKD stage 3–5 before study entry, which was determined by diagnostic codes and eGFR measurements (following our definition of CKD onset).

We also defined a cohort of patients with complete follow-up data, consisting of patients who either developed CKD in the study period or had at least 5 years of follow-up. We used this cohort to validate models derived with logistic regression, which requires complete follow-up data.

#### Predictors and missing data

We used Read codes retrieved from clinicalcodes.org [28] to extract clinical and laboratory variables from the SIR database. Clinicalcodes.org is a repository of Read codes used in previously published articles; we used Read codes from five studies [29–33] (see Additional file 1 for full list of adopted Read codes). For comorbidities, such as hypertension and peripheral vascular disease, we identified any related diagnostic Read code before the patient’s study entry date. If the type of diabetes was not specified in the diagnostic code or contradicting codes were present (i.e. diabetes type 1 and type 2 for the same patient), we assigned ‘type 1’ to patients with the first diabetes code before 35 years of age, and ‘type 2’ to all other diabetes patients. For medications, such as nonsteroidal anti-inflammatory drugs or hypertensive medications, we looked for at least two prescriptions in the 6 months prior to entry date. Finally, for laboratory tests, we selected the most recent result within 12 months before the entry date.

Since more than 90 % of the population in Salford is of White British ethnicity [34], we considered patients without a recorded ethnicity code as White British. We imputed values for predictors using multiple imputation by chained equations with 10 iterations to minimise the effect of selectively ignoring those with any missing data (using the *mice* package in R [35]).

### Data analysis

We implemented models developed by logistic and Cox proportional hazards (CPH) regression formulas using published coefficients and intercept or baseline hazard provided. For the QKidney models [36] we used the information from svn.clinrisk.co.uk/opensource/qkidney – a web-based calculator written in C (re-coded in R language as per Additional file 2). For simplified scoring systems, we computed the total simplified score for each patient in our dataset. In addition, if the original model was a logistic regression and the intercept was not reported, we estimated it from information about CKD prevalence and predictors summary measures (mean for continuous variables and prevalence for binary variables) in the development population.

We assessed the performance of the models and the associated simplified scoring systems in terms of discrimination and calibration. Discrimination is the ability of a model to distinguish between patients who do or do not develop CKD. Discrimination was assessed by calculating the area under receiving operating characteristic curve (AUC) and Harrell’s c-index [37–39]; 95 % confidence intervals (CIs) for the AUC and c-index were calculated from 500 bootstrap iterations. We evaluated calibration by calculating the mean absolute prediction error (MAPE), calibration slope, and by calibration plots. MAPE is the average difference in predicted and observed onset of CKD, expressed by a number between 0 and 1, with values closer to 0 indicating better performance [40] (see Additional file 3 for details). Calibration slopes are regression slopes of linear predictors fitted to the external validation dataset [41]. The optimal value is 1, with values smaller than 1 reflecting overfitting of the model. Calibration plots compare mean predicted risks with mean observed outcomes for subgroups with similar predicted risks. A model is considered to be well calibrated if the plot follows the 45° line from the lower left corner to the upper right corner of the plot. In our analysis, we created calibration plots using the R package PredictABEL [42].

For the simplified scoring systems, we compared sensitivity, specificity and positive predictive value (PPV) obtained by using the decision-making threshold that was reported in the original publications, as well as using the optimal threshold for our study population as calculated with Youden’s method [43]. If a study did not present any risk score or we could not use the proposed simplified score because of more than one missing predictor in our dataset, sensitivity, specificity and PPV were evaluated for the full model instead.

To interpret the performance of included models we used the framework for external validation from Debray et al. [44]. Therefore, we assessed the extent to which the case-mix of the development datasets and our validation dataset were similar, by comparing the mean linear predictor of models in the two cohorts. Since individual patient data of the development datasets were not publicly available, the mean linear predictor was calculated as the sum of the intercept and the product of model coefficients and predictors’ prevalence (for binary variables) or mean (for continuous variables) provided in the summary statistics of original studies. In order to assess how accurate the mean linear predictor calculation based on the summary statistics was, in our validation dataset we also calculated the mean linear predictor by calculating the mean and standard deviation (SD) of the linear predictor from the individual patient data.

Finally, to evaluate the clinical impact of implementing the models in practice as screening tools, we performed two analyses. First, we performed decision curve analysis evaluating how different threshold probabilities alter the false-positive and false-negative rate expressed in terms of net benefit [45]. When carrying out a head-to-head comparison of different prediction models on the same population, the interpretation is straightforward – at each clinically relevant probability threshold, the model that has the highest net benefit is preferable. Models are also compared to the extreme choices of designating all and no patients at high risk of developing CKD. Second, for each model, we evaluated the potential implementation of a CKD prevention high-risk approach [46] based on the model’s prediction by calculating the proportion of observed CKD cases in our dataset within the highest tenth of predicted risk (i.e. the 10 % of patients with highest predicted risks).

Data manipulation and statistical analyses were performed using R software (www.r-project.org).

### Sensitivity analyses

We performed several sensitivity analyses. First, since the risk of developing CKD in the asymptomatic general population is low [47], we also validated each of the models in patients with established CKD risk factors at entry date. Following the UK National Institute for Clinical Excellence (NICE) guidelines on early detection of CKD [11], these risk factors were use of calcineurin inhibitor drugs, lithium, or nonsteroidal anti-inflammatory drugs; diabetes mellitus; hypertension; acute kidney injury in the previous 2 years; history of cardiovascular disease, renal calculi or prostatic hypertrophy, systemic lupus erythematosus, or haematuria; and family history of kidney disease. Second, as most models in our study used a single measured renal impairment to define CKD, we repeated the analysis while using a more inclusive definition of CKD onset as the presence of a CKD 3–5 diagnostic code or a single eGFR measurement below 60 mL/min/1.73 m^2^. Third, we considered patients who died during follow-up as if they developed CKD, because mortality is frequently attributable to CKD and most risk prediction models do not account for death as a competing risk. Fourth, we calculated eGFR by using the Chronic Kidney Disease Epidemiology Collaboration (CKD-EPI) formula [48] and repeated our main analysis (e.g. CKD defined as impaired eGFR for at least 3 months or CKD 3–5 diagnostic code). Fifth, we repeated our main analysis by using a prediction horizon of 4 instead of 5 years. Finally, we repeated the analyses omitting individuals with any missing observation.

## Results

### CKD prediction models included for external validation

**Fig. 1:**
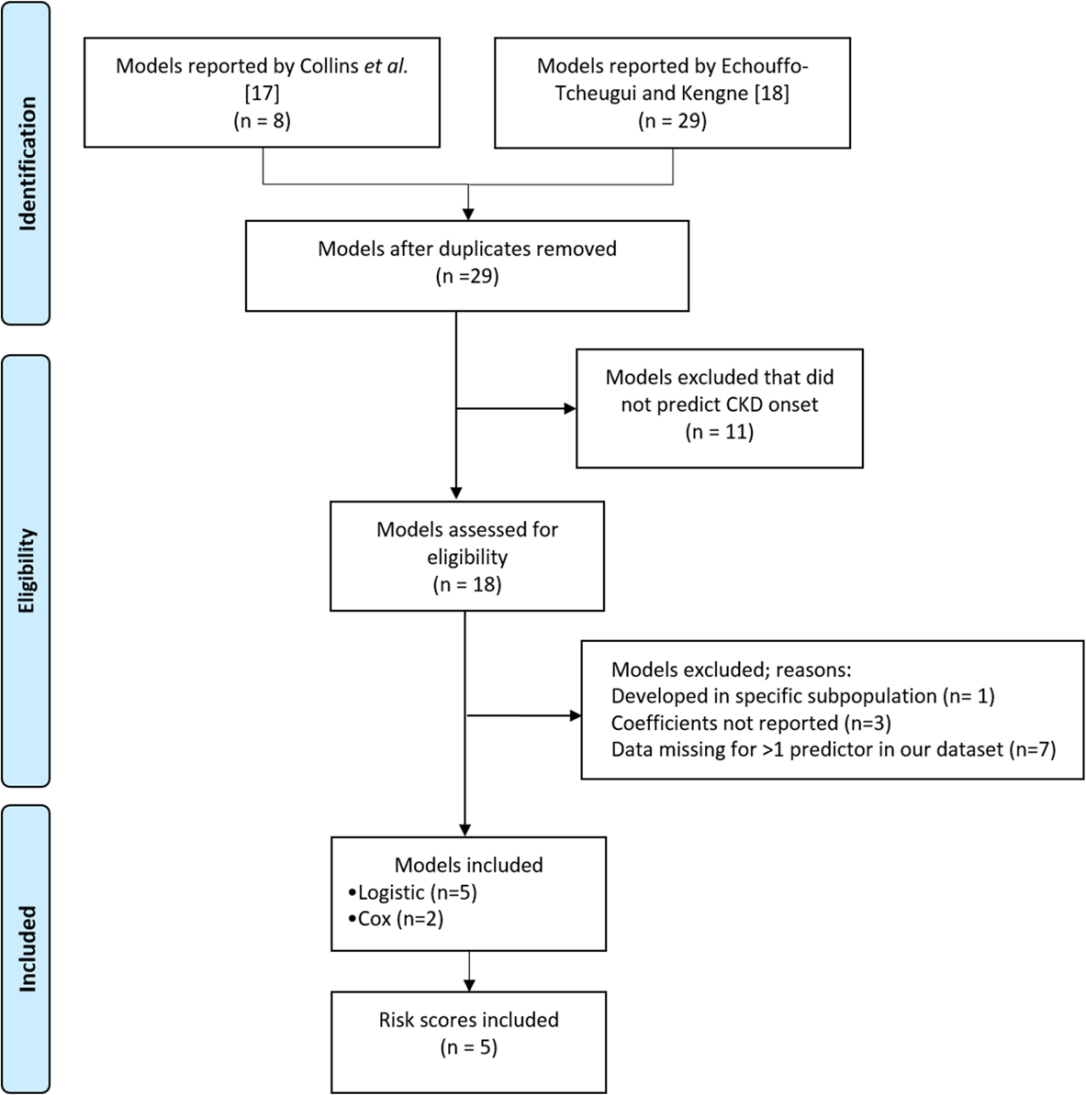
Procedure to identify and select CKD prediction models

Figure 1 depicts the model inclusion process. Of the 29 models identified by Collins et al*.* [14] and Echouffo-Tcheugui and Kengne [15], 18 were developed with the aim of predicting CKD onset. We excluded three models because of incomplete reporting of regression models (regression coefficients not fully reported) in the original paper [49] and one model because it was developed in a specific sub-population (namely HIV patients) [20]. We excluded a further seven models for which we had more than one missing predictor in our dataset, including missing data for eGFR, urinary excretion, and c-reactive protein [50]; missing post-prandial glucose, proteinuria and uric acid [51]; missing eGFR and quantitative albuminuria [52], and finally, we excluded two models because of missing eGFR and low levels of high-density lipoprotein cholesterol [52, 53], respectively. The final set consisted of seven models (five logistic regression models and two CPH regression models) and five simplified scoring systems [36, 51–56]. Table 1 describes the details of the included models, and Additional file 3: Tables S1, S2 and S3 provide the population characteristics of the development datasets, the regression coefficients, and the simplified scoring systems.

**Table 1:** Details of studies developing CKD prediction models that were included for external validation

Authors [ref] Publication year	Study design/Study context Study period	Ethnicity Age range	Population size Number (%) of CKD cases	Type of models Time horizon	Handling of missing values Method of internal validation	Definition of CKD	Predictors in model
Bang et al. [54] 2007	Cross-sectional population-based survey/Screening programme 1999-2002	US, mixed 20–85 years	8530 601 (7.5 %)	Logistic 2 years	Excluded Random split-sample	At least one eGFR measurement < 60^a^	Age, sex, anaemia, proteinuria^a^, hypertension, diabetes mellitus, history of cardiovascular disease, history of heart failure, peripheral vascular disease
Chien et al. [51] 2010	Prospective cohort study/ Secondary care 2003	Taiwan, Chinese 51.2 years (mean)	5168 190 (3.7)	Cox 4 years	NR NR	At least one eGFR measurement < 60^a^	Age, BMI, diastolic blood pressure, type 2 diabetes, history of stroke
Hippisley-Cox and Coupland (QKidney®) [36] 2010	Prospective cohort population based/Primary care 2002-2008	UK, mixed 35–74 years	1,591,884 23,786 (1.5 %)	Cox 5 years	Multiple imputation Random split-sample	At least one eGFR measurement < 45^a^, kidney transplant; dialysis; nephropathy diagnosis; proteinuria	Age, ethnicity, deprivation, smoking, BMI, systolic blood pressure, diabetes mellitus, rheumatoid arthritis, cardiovascular disease, treated hypertension, congestive cardiac failure, peripheral vascular disease, NSAID use, and family history of kidney disease
Kshirsagar et al. [53] 2008	Prospective cohort study/ Community-based 1987-1989	US, white and black 45–64 years	9470 1605 (16.9 %)	Logistic 9 years	NR Random split sample	At least one eGFR measurement < 60^a^	Age, sex, anaemia, hypertension, type 2 diabetes mellitus, history of cardiovascular disease, history of heart failure, peripheral vascular disease
Kwon et al. [55] 2012	Cross-sectional survey/ Population-based 2007-2009	Korean, Asian ≥19 years	6565 100 (1.5 %)	Logistic 1 year	Excluded Split sample	At least one eGFR measurement < 60^a^	Age, sex, anaemia, proteinuria^a^, hypertension, type 2 diabetes mellitus, history of cardiovascular disease
O’Seaghdha et al. [52] 2011	Prospective cohort study/ Population-based 1995-2008	US white 45–64 years	2490 229 (9.2 %)	Logistic 10 years	Excluded Bootstrap	At least one eGFR measurement < 60^a^	Age, hypertension, diabetes mellitus
Thakkinstian et al. [56] 2011	Cross-sectional survey/ Community-based NR	Thailand-Asian ≥ 18 years	3459 606 (17.5 %)	Logistic 1 year	NR Bootstrap	At least one eGFR measurement < 90^a^	Age, hypertension, diabetes mellitus, kidney stones

BMI, body mass index; CKD, chronic kidney disease; eGFR, estimated glomerular filtration rate, NR, not reported; NSAID, non-steroidal inflammatory drugs; US, United States

^a^Predictor not included in external validation due to missing data in our dataset

All models were developed outside the UK, with the exception of QKidney® [36] (www.qkidney.org), which was developed on a large population from England and Wales selected from general practices using the EMIS EHR. All included models used a different definition of CKD, but the majority used an older definition based only on one impaired eGFR measurement. Time horizons in original papers were different to our 5-year definition, with the exception of QKidney® [36], which, however, allowed other time horizon options (1-, 2-, 3- and 4-year). For three models, the prediction time horizon was not specified [54–56]. However, we could derive from study duration and data collection procedures in the original publications that the time horizons were 1 [56], 2 [54] and 9 [54] years, respectively. For the remaining models, the reported time horizons were between 4 and 10 years [51, 52, 54].

Predictors included in the models were largely based on known CKD risk factors (hypertension, diabetes mellitus, or history of cardiovascular disease). The only biomarkers included were systolic and diastolic blood pressure, and body mass index. Multiple imputation of missing values was applied to these variables, along with deprivation, haemoglobin (i.e. to calculate presence of anaemia) and smoking. In these predictors, missing values ranged from 1.8 % to 70.0 %, with a median value of 46.0 %. Conversely, we excluded proteinuria as a predictor from our analyses due to 94.6 % missing data (Table 2); therefore, the models by Bang et al. [54] and Kwon et al. [55] had one missing predictor. Finally, three of the included models, which derived a simplified scoring system [53, 55, 57], did not report the intercept of their underpinning logistic regression model, and therefore we estimated the intercepts from the prevalence of CKD and predictors’ summary statistics in the original studies.

**Table 2:** Patients with complete and incomplete follow-up data stratified for CKD onset; values are numbers (%) unless indicated otherwise

Parameters		No CKD				CKD	
		Patients with complete follow-up		Patients with incomplete follow-up			
			Missing		Missing		Missing
Included patients		156,615	None	172,361	None	6038	None
Died before developing CKD		719 (0.5)	None	6941 (4)	None	/	None
Follow-up (mean, SD)		5.6 (0.2)	None	5.4 (0.7)	None	2.6 (1.7)	None
Age (mean, SD)		42.1 (16.7)	None	42.7 (17.3)	None	70.3 (12.5)	None
Female sex		82,883 (52.9)	None	89,389 (51.9)	None	3452 (57.2)	None
Townsend index (mean, SD)^e^		1.6 (3.5)	2900 (1.9)	1.6 (3.4)	3244 (1.9)	1.4 (3.4)	47 (0.8)
Ethnicity	Not recorded	55,586 (35.6)	Not applicable^d^	61,220 (35.6)	Not applicable^d^	2014 (33.4)	Not applicable^d^
	White	90,443 (57.8)		99,243 (57.7)		3889 (64.5)	
	Other	10,586 (6.8)		11,898 (6.9)		135 (2.2)	
Smoking^e^	Non-smoker	66,769 (48.8)	19,901 (12.7)	72,137 (48.4)	23,296 (13.5)	2167 (37.7)	292 (4.8)
	Ex-smoker	29,980 (21.9)		33,097 (22.2)		2475 (43.1)	
	Light smoker (1–9 cg/day)	11,072 (8.1)		12,128 (8.1)		344 (6)	
	Moderate smoker (10–19 cg/day)	16,951 (12.4)		18,472 (12.4)		413 (7.2)	
	Heavy smoker (≥ 20 cg/day)	11,942 (8.7)		13,231 (8.9)		347 (6)	
BMI, kg/m^2^ (mean, SD)^e^		26.6 (6)	33,717 (21.5)	28 (6.1)	38,628 (22.4)	28.4 (6)	518 (8.6)
Diastolic blood pressure, mmHg (mean, SD)^e^		76.9 (9.8)	75,616 (48.3)	78.9 (10.2)	85,075 (49.4)	75.8 (10.2)	1164 (19.3)
Systolic blood pressure, mmHg (mean, SD)^e^		128.2 (15.8)	75,602 (48.3)	130.5 (16.7)	85,058 (49.3)	136.3 (16.7)	1166 (19.3)
eGFR, mL/min/1.73 m^2^ (mean, SD)		83.7 (9.4)	118,912 (75.9)	82.5 (9.4)	131,103 (76.1)	69.4 (11.3)	1828 (30.3)
Hb, g/dL^e^		13.9 (1.6)	110,723 (70.7)	13.8 (1.6)	122,430 (71)	13.4 (1.7)	2530 (41.9)
Proteinuria^a,b^		751 (0.5)	149,234 (95.3)	18 (0.2)	164,097 (95.2)	236 (3.9)	4665 (77.3)
Quantitative albuminuria^b,c^		129 (0.1)	152,266 (97.2)	4 (0)	167,482 (97.2)	62 (1)	5167 (85.6)
HDL cholesterol level^b^, mg/dL (mean, SD)		25.9 (7.9)	122,477 (78.2)	26.7 (7.9)	135,066 (78.4)	25.7 (7.8)	2413 (40)

BMI, body mass index; cg, cigarettes; CKD, chronic kidney disease; eGFR, estimated glomerular filtration rate; HDL, high-density lipoprotein; MDRD, modification of diet in renal disease; SD, standard deviation

^a^Albumin:creatinine ratio >30 mg/mmol or albumin concentration >200 mg/L, or diagnostic code

^b^Variable excluded as predictor from external validation due to >70 % missing values

^c^Albumin:creatinine ratio >30 mg/mmol

^d^Patients without recorded ethnicity were considered as white (see Methodssection)

^e^Multiple imputation applied to missing values

### Study population characteristics

**Fig. 2:**
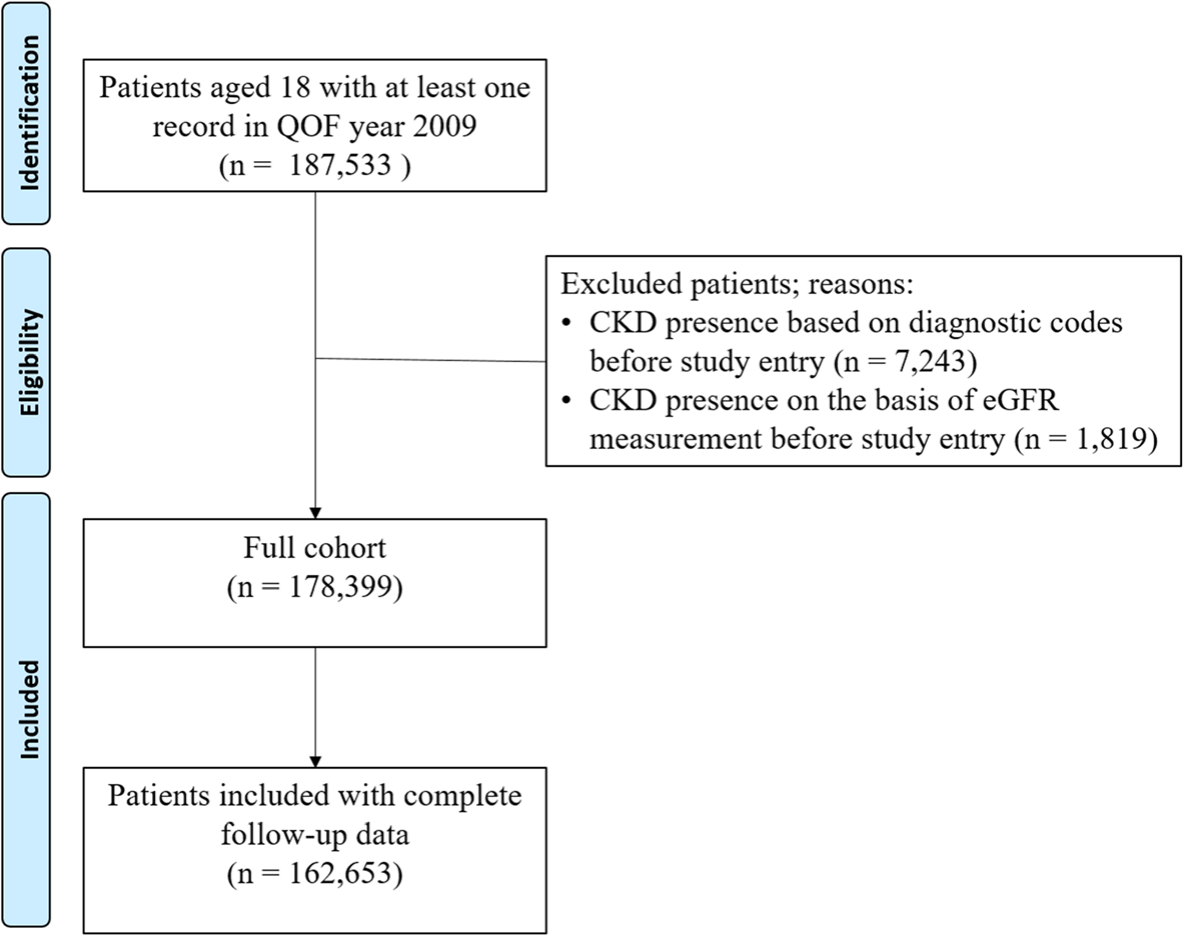
Cohort selection

Figure 2 shows the cohort selection process. There were 187,533 adult patients with at least one record in the financial year 2009 in our database, of which 178,399 remained after applying our exclusion criteria, with 6941 patients (3.9 %) that died before developing CKD. There were 162,653 patients (91.2 %) who had complete follow-up data. Overall, there were 6038 incident cases of CKD during the study period. Tables 2 and 3 describe the characteristics of cohorts with complete and incomplete follow-up.

**Table 3:** Prevalence of CKD risk factors (as expressed in NICE guidelines) stratified for CKD onset; values are numbers (%) unless indicated otherwise

CKD risk factors	No CKD		CKD (*n* = 6038)
	Patients with complete follow-up (*n* = 156,615)	Patients with incomplete follow-up (*n* = 172,361)	
Hypertension^a^	22,074 (14.1)	24,971 (14.5)	3554 (58.9)
Hypertensive treatment^b^	22,122 (14.1)	24,769 (14.4)	3655 (60.5)
Type 1 diabetes mellitus^a^	703 (0.4)	740 (0.4)	36 (0.6)
Type 2 diabetes mellitus^a^	5574 (3.6)	6383 (3.7)	1221 (20.2)
History of cardiovascular disease^a^	11,096 (7.1)	13,407 (7.8)	2182 (36.1)
History of heart failure^a^	743 (0.5)	1088 (0.6)	387 (6.4)
History of stroke^a^	1875 (1.2)	2538 (1.5)	509 (8.4)
Peripheral vascular disease^a^	2127 (1.4)	2532 (1.5)	331 (5.5)
Kidney stones^a^	751 (0.5)	814 (0.5)	64 (1.1)
Rheumatoid arthritis^a^	1321 (0.8)	1512 (0.9)	142 (2.4)
Systemic lupus erythematosus^a^	99 (0.1)	104 (0.1)	8 (0.1)
Family history of kidney disease^a^	25 (0)	28 (0)	3 (0)
NSAID use^b^	5101 (3.3)	5389 (3.1)	402 (6.7)
Acute kidney injury in the last 2 years	1975 (1.3)	2633 (1.5)	413 (6.8)
Prostatic hypertrophy^a^	967 (0.6)	1143 (0.7)	173 (2.9)
Haematuria^a^	3176 (2)	3574 (2.1)	341 (5.6)
Lithium use^b^	150 (0.7)	219 (0.1)	52 (0.9)
Tacrolimus use^b^	4 (0)	5 (0)	2 (0)
Cyclosporin use^b^	12 (0.1)	20 (0)	6 (0.1)

NSAIDs, Non-steroidal anti-inflammatory drugs; SD, standard deviation

^a^Based on diagnostic Read codes

^b^At least two prescriptions in the 6 months before entry date

### External validation

**Fig. 3:**
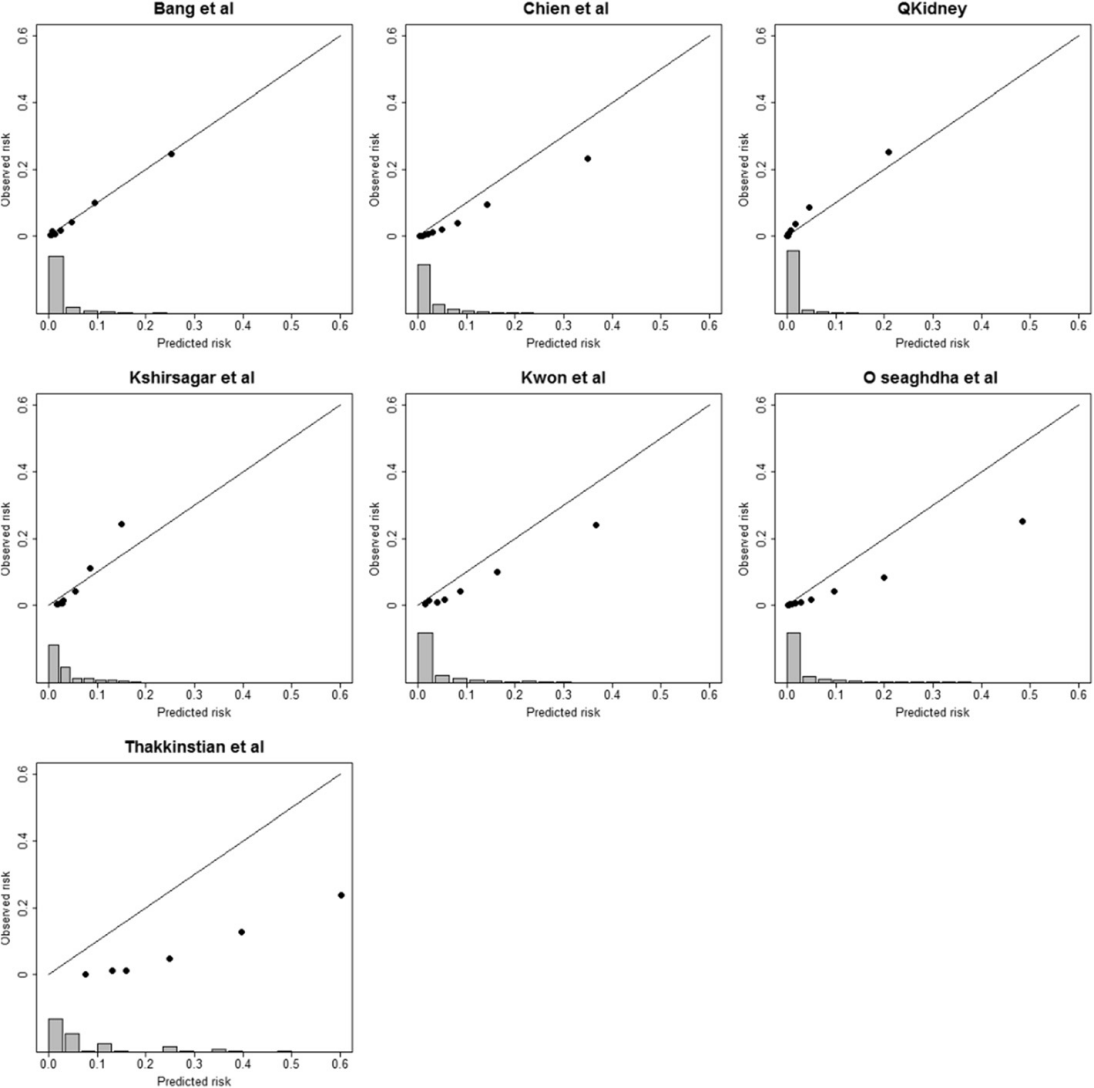
Calibration plot of predicted and observed risk for the cohort of patients with complete follow-up. On the bottom a rug plot in the form of histogram shows the distribution of the predicted values

Table 4 presents the results of the external validation, namely discrimination and calibration. AUC values ranged from 0.892 (95 % CI, 0.888–0.985) to 0.910 (95 % CI, 0.907–0.913) for patients with complete follow-up data, and the c-index values for the two CPH models on the full cohort were 0.888 (95 % CI, 0.885–0.892) [51] and 0.900 (95 % CI, 0.897–0.903) [36], respectively. Simplified scores showed similar performance to the models from which they were derived. MAPE was below 0.1 for all models, with the only exception of Thakkinstian et al. [56], for which the MAPE was 0.179 (standard deviation (SD), 0.161). Calibration plots (Fig. 3) and related calibration slopes (Table 4) on the complete follow-up data showed similar figures to the MAPE analysis. Thakkinstian et al. [56] confirmed a tendency for over-predicting risk with a calibration slope of 0.44 (95 % CI, 0.43–0.45). Conversely, the only models that were well-calibrated to our population were the ones by Bang et al. [54] and QKidney® [36] with calibration slope values of 0.97 (95 % CI, 0.96–0.98) and 1.02 (95 % CI, 1.01–1.04), respectively. All other models over predicted risks (i.e. calibration slopes ranging between 0.53 [ 95 % CI, 0.52–0.53] and 0.68 [ 95 % CI, 0.67–0.69] ), with the exception of the model by Kshirsagar et al. [53], which predicted lower risk and had a calibration slope of 1.74 (95 % CI, 1.72–1.76).

**Table 4:** Discrimination, MAPE and calibration slopes of included models in patients with complete follow-up data (all models and risk scores) and in the full validation cohort (Cox proportional hazards regression models only)

	Study	Patients with complete follow-up (*n* = 162,653)			Full validation cohort (*n* = 178,399)	
		AUC (95 % CI)	MAPE (SD)^a^	Calibration slope (CI)	c-index (95 % CI)	MAPE (SD)^a^
Models	Bang et al. [54]	0.899 (0.895–0.903)	0.063 (0.162)	0.97 (0.96–0.98)	NA	NA
	Chien et al. [51]^b^	0.898 (0.895–0.901)	0.081 (0.162)	0.65 (0.64–0.65)	0.888 (0.885–0.892)	0.085 (0.166)
	QKidney® [36]^b^	0.910 (0.907–0.913)	0.05 (0.166)	1.02 (1.01–1.04)	0.900 (0.897–0.903)	0.052 (0.165)
	Kshirsagar et al. [53]	0.896 (0.892–0.900)	0.068 (0.164)	1.74 (1.72–1.76)	NA	NA
	Kwon et al. [55]	0.899 (0.895–0.902)	0.086 (0.158)	0.68 (0.67–0.69)	NA	NA
	O’Seaghdha et al. [52]	0.907 (0.904–0.911)	0.089 (0.169)	0.53 (0.52–0.53)	NA	NA
	Thakkinstian et al. [56]	0.892 (0.888–0.985)	0.179 (0.161)	0.44 (0.43–0.45)	NA	NA
Simplified Scores	Bang et al. [54]	0.895 (0.891–0.899)	NA	NA	NA	NA
	Chien et al. [51]	0.880 (0.876–0.883)	NA	NA	NA	NA
	Kshirsagar et al. [53]	0.891 (0.887–0.895)	NA	NA	NA	NA
	Kwon et al. [55]	0.895 (0.891–0.898)	NA	NA	NA	NA
	Thakkinstian et al. [56]	0.869 (0.864–0.873)	NA	NA	NA	NA

AUC, area under receiver operating characteristic curve; eGFR, estimated glomerular filtration rate; NA, not applicable; SD, standard deviation; CI, confidence interval.

^a^Calculated as mean difference between observed and predicted CKD cases

^b^Cox proportional hazard regression model

Table 5 reports the PPV, sensitivity and specificity for each of the simplified scoring systems. In this analysis we included the full QKidney® [36] model as it does not have an associated simplified scoring system. We also included the full O’Seaghdha et al. [52] model because we could not implement their scoring system: multiple predictors had 70 % or more missing values in our dataset. For two scoring systems (Chien et al. [51] and Thakkinstian et al. [56]), the best threshold in our population was different than the threshold proposed in the development study. For QKidney® [36] and O’Seaghdha et al. [52], who did not report a threshold in the development study, the optimal threshold in our population was 0.017 (SD, 0.002) and 0.086 (SD, 0.010), respectively. In terms of performance, models showed similar performance, with a PPV, sensitivity and specificity of approximately 0.145, 0.86 and 0.80, respectively.

**Table 5:** Positive predictive value, sensitivity and specificity for simplified scoring systems when applying to the threshold that was proposed in the development study and best threshold on our dataset, calculated using the Youden’s method [43]

Study		Threshold (SD)	PPV (SD)	Sensitivity (SD)	Specificity (SD)
Bang et al. [54]	Proposed	4	0.146 (0.002)	0.865 (0.004)	0.805 (0.001)
	Best	4	0.146 (0.002)	0.865 (0.004)	0.805 (0.001)
Chien et al. [51]	Proposed	7	0.106 (0.001)	0.916 (0.003)	0.701 (0.001)
	Best	8	0.133 (0.002)	0.863 (0.004)	0.783 (0.001)
QKidney® [36]	Proposed	NR	NA	NA	NA
	Best	0.017 (0.002)	0.147 (0.006)	0.870 (0.012)	0.805 (0.012)
Kshirsagar et al. [53]	Proposed	3	0.143 (0.002)	0.872 (0.004)	0.799 (0.001)
	Best	3	0.143 (0.002)	0.872 (0.004)	0.799 (0.001)
Kwon et al. [55]	Proposed	4	0.147 (0.002)	0.862 (0.004)	0.807 (0.001)
	Best	4	0.147 (0.002)	0.862 (0.004)	0.807 (0.001)
O’Seaghdha et al. [52]	Proposed	NA	NA	NA	NA
	Best	0.086 (0.010)	0.138 (0.007)	0.885 (0.015)	0.786 (0.015)
Thakkinstian et al. [56]	Proposed	5	0.071 (0.001)	0.936 (0.003)	0.529 (0.001)
	Best	6	0.140 (0.002)	0.861 (0.004)	0.796 (0.001)

PPV, positive predictive value; NR, Not reported; NA, not applicable; SD, standard deviation

Note: As QKidney® does not have any associated score in the original publication, we reported results for the full model. O’Seaghdha et al. [52] reported a simplified score system; however, this could not be used in our population because of missing predictors. Therefore, we calculated performance for the full model instead

The distributions of the linear predictors in the development datasets and the validation dataset, calculated as proposed by Debray et al. [44], are shown in Table 6. For all models, the mean of the linear predictor in the validation dataset was lower than in the development datasets: we found mean differences between 0.2 and 0.6, except for the model of Thakkinstian et al. [56], which had a difference of 1.5. There were few differences between the mean linear predictors computed on our dataset using summary statistics compared with individual patient data.

**Table 6:** Mean linear predictor, calculated in development datasets and in our validation dataset (patients with complete follow-up data only)

	Study	Development dataset	Validation dataset, patients with complete follow-up (*n* = 162,653)	
		Mean linear predictor (from summary statistics)	Mean linear predictor (from summary statistics)	Mean linear predictor (SD) (from individual patient data)
Models	Bang et al. [54]	−3.9	−4.2	−4.2 (1.4)
	Chien et al. [51]	0.1	−0.5	−0.5 (1.5)
	QKidney® [36]	−0.1	−0.3	−0.1 (1.9)
	Kshirsagar et al. [53]	−3.0	−3.5	−3.5 (0.8)
	Kwon et al. [55]	−3.0	−3.4	−3.3 (1.2)
	O’Seaghdha et al. [52]	−1.6	−1.8	−1.8 (0.9)
	Thakkinstian et al. [56]	−2.3	−3.8	−3.8 (1.9)

The threshold probability associated with the highest tenth of predicted risk varied from 0.0692 for QKidney® [36] to 0.4256 for the model developed by Thakkinstian et al. [56]. When applying these thresholds to select the 10 % of patients with highest predicted risks, QKidney® [36] identified 64.5 % of all patients that developed CKD during the study period. Proportions for the other models ranged from 48.0 % for the model from Thakkinstian et al. [56] to 64.0 % for the model of O’Seaghdha et al. [52].

**Fig. 4:**
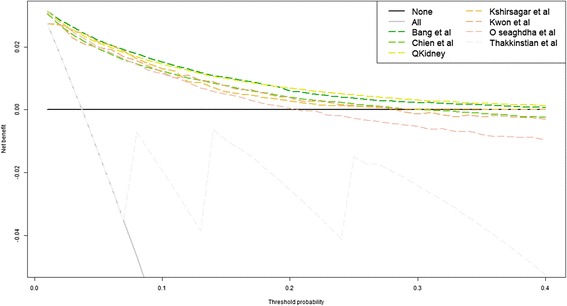
Decision curve analysis for the cohort of patients with complete follow-up

Decision curves for the cohort of patients with complete follow-up are presented in Fig. 4. The models by Bang et al. [54] and QKidney® [36] had the best performance. At predicted probability thresholds lower than 0.5, their net benefit was greater than all other models and greater than strategies labelling all patients at high risk (black line) or none at high risk (grey line). For predicted probability thresholds greater than 0.5, Bang et al. [54] and QKidney® [36] were equivalent to the choice of not labelling any patient as high CKD risk (grey line).

### Sensitivity analyses

The sensitivity analysis conducted on patients with CKD risk factors showed comparable calibration and MAPE (Bang et al. [54] and QKidney® [36] were the only well-calibrated models), with an overall decrease in discrimination of about 0.1 (Additional file 3: Table S4) compared to our main analysis. Specifically, AUC values on patients with complete follow-up ranged from 0.756 (95 % CI, 0.749–0.762) to 0.801 (95 % CI, 0.795–0.808), while the c-index values for the two Cox regression models were 0.755 (95 % CI, 0.749–0.761) [51] and 0.775 (95 % CI, 0.769–0.781) [36], respectively. The performance of the simplified scoring systems was worse compared to the models from which they were derived.

The sensitivity analysis in which CKD was defined by the presence of only one eGFR measurement lower than 60 mL/min/1.73 m^2^ or a diagnostic code for CKD 3–5 led to a higher prevalence of CKD onset (5.2 %, *n* = 8854), with an overall predictive model performance that slightly decreased (Additional file 3: Table S5), especially in terms of calibration. CKD onset prevalence was also higher (3.9 %, *n* = 6988) when we calculated eGFR by using the CKD-EPI formula, with an increase in absolute numbers of approximately 1000 cases and an average age in this group of 76 years (SD, 8.1). Overall performance was similar to our main analysis, and only the model by Bang et al. [54] was well-calibrated in this sensitivity analysis (Additional file 3: Table S8). As expected, we witnessed an increase in CKD onset prevalence (7.6 %, *n* = 13,652) when we counted patients that died during follow-up as if they developed CKD; however, that did not lead to changes in discriminative performance of the models (Additional file 3: Table S6). Conversely, calibration improved for all models that were over-predicting CKD in our main analysis. In the analysis restricted to patients with complete data on all predictors we found an overall decrease in performance of about 0.08 for AUCs and c-index (Additional file 3: Table S7), while the sensitivity analysis that used a 4-year time horizon showed similar discriminative performance to our main analysis, but worse calibration for all models except QKidney® (Additional file 3: Table S9).

## Discussion

We externally validated and compared seven published models for the prediction of CKD onset [14, 15], using a recent 5-year window with well-studied EHR data, typical of UK NHS primary care and chronic disease management. All models discriminated well between patients who developed CKD compared with those who did not. Five models had an associated simplified scoring system, each of which had a similar performance to its parent model. Only two models were well-calibrated to the risk levels in our population [36, 54]. Among the 10 % of patients with highest predicted risks, 48.0 % to 64.5 % actually developed CKD.

Two key strengths of this study are (1) its large sample size and (2) its cohort being based on a geographically-defined population rather than tied to a particular EHR, which minimizes selection bias at enrolment. In addition, whilst five out of seven models had already been externally validated [17, 36, 51, 54, 55, 58] and two had been mutually compared [17], our study is the first comprehensive head-to-head comparison of multiple CKD prediction models on a large independent population.

Three previous UK-based studies [36, 58, 59] have externally validated QKidney® [36] and reported a c-statistic of 0.87, good calibration and similar proportions of identified CKD cases among the 10 % of patients with highest predicted risks. Although each study externally validated QKidney® [36] using UK primary care EHR data, our study extended the validation. Collins et al. [59] and Hippisley-Cox and Coupland [36, 58] adopted the same inclusion criteria as in the original development study [36] (i.e. patients aged between 35 and 74 years), CKD definition (i.e. eGFR measurement <45 mL/min/1.73 m^2^, kidney transplant, dialysis, nephropathy diagnosis and proteinuria) and stratification by sex. However, the present study included all adults (aged 18 years and over) and used a more robust definition of the outcome.

A previous study compared the models from Chien et al*.* [51] and Thakkinstian et al. [56] in mixed-ancestry South Africans [17]. The present study found that these models underestimated CKD risk in this population, while in our external validation both models over-predicted CKD risk. A likely explanation is the difference in CKD onset prevalence between the development cohorts, the cohort from the Mogue et al. [17] dataset, and our cohort. Specifically, the study population from Mogue et al. [17] had a much higher prevalence of CKD cases than these development cohorts, while our study population had a lower prevalence.

The included prediction models and simplified scoring systems had remarkably good discriminative ability in our dataset, with better performance than in most of the original studies. This is, on the one hand, surprising because models usually perform similarly or worse in external validation. On the other hand, we used a more robust definition of CKD, requiring impaired eGFR (eGFR < 60 mL/min/1.73 m^2^) for at least 3 months, rather than the one used in most of the original studies [51–55], which looks at CKD measurements in isolation. The latter definition inflates incidence of CKD diagnosis [60] and therefore leads to a poorer signal-to-noise ratio and a decrease in model performance [61], as shown in our sensitivity analysis (Additional file 3: Table S5). Another advantage of our definition, which is based on the international Kidney Disease: Improving Global Outcomes (KDIGO) guidelines [62], is that it is closer to the definition of CKD currently used in UK clinical practice. Along the same lines, we used the MDRD formula to calculate eGFR, which is currently used in UK clinical practice. We also performed a sensitivity analysis to investigate whether using the CKD-EPI formula [48] would have led to different results, which confirmed the findings from Carter et al. [63] that the CKD-EPI formula calculates lower eGFR values than the MDRD formula for older patients.

In the complete case analysis, and in the analysis restricted to patients with established CKD risk factors, there was a marked decrease in discriminative performance. In both cases, further to the decrease in sample size, a plausible explanation is that these analyses increased the differences in case-mix between development and validation datasets. The complete case analysis considers only patients without missing predictors, who are more likely to have had healthcare contacts related to their disease. As in the cohort with established CKD risk factors, this excludes a large group of healthy patients, and thus leads a quite different population than the one for which the models were developed. Based on our findings it seems that a different model is needed for patients with established CKD risk factors. Such a model could use other information that is not routinely available in the majority of the low-risk population, like creatinine levels.

We observed an over-prediction of CKD risk by the majority of models, which can be explained largely by differences in case-mix between our validation cohort and the development populations. First, the incidence of CKD in most development datasets was higher than in our validation cohort. As a consequence, the baseline CKD risks calculated (i.e. model intercepts) in the development datasets were too high for our population. Furthermore, as the mean linear predictor analysis showed, our population appeared to be healthier (i.e. lower mean predictor values) than the populations used in the development studies. We also found, in some models, unexpectedly large coefficients for some covariates. For example, three of the included models [53–55] had coefficients for covariates such as anaemia or peripheral vascular disease that were either comparable or larger than more well-established CKD risk factors like diabetes or hypertension. Finally, another possible explanation of the models’ poor calibration is the adoption of a slightly different definition for some predictors in this study, in concordance to the ones used in the NHS, when compared to the original studies.

No calibration problems were found for the models by Bang et al. [54] and QKidney® [36]. However, we left out an important predictor from the model by Bang et al. [54], proteinuria, because it was missing from our dataset. Because the model is well calibrated now, we expect that it would have over-predicted risks if proteinuria had been present. QKidney® [36] was originally developed in the UK primary care (England and Wales), and it was the only model for which the analysed time horizon (5 years) was the same as in the development paper. Therefore, a good calibration was expected. This was confirmed by the fact that we obtained similar mean linear predictors in our dataset to the ones reported in the original development study (Table 6).

Overall, the only model that could conceivably be applied in our population without recalibration was QKidney® [36]. QKidney® consistently outperformed all the other models in terms of both discrimination and calibration, and its performance is comparable to existing validation studies [36, 58, 59]. The model could be used via the web calculator (www.qkidney.org) or directly integrated into EHRs.

From a methodological perspective, there is room for improvement in CKD prediction modelling. First, future studies should consider to use the CKD definition provided by the international KDIGO guidelines [62]. This should also be used to re-estimate the CKD risk prediction models already available. Second, none of the models included in our analysis accounted for death as a competing risk. We recommend that authors of future models use methodologies [64, 65] to do so. Third, authors should take advantage of the new opportunities offered by EHR databases to develop and validate future CKD prediction models [66]. Particularly, besides the possibility of accessing larger sample sizes and to have more predictors, EHRs give the opportunity of observing repeated measurements and account for changes over time of patient’s relevant conditions and biomarkers [66, 67]. This is particularly important in CKD, where comorbidities and biomarkers like creatinine play a key role.

Our study has several limitations. First, we excluded 11 models identified from the two reviews [14, 15] because they included variables not present in our data. However, these models were qualitatively less applicable to our prediction population/context than those included. Second, we removed proteinuria from the models by Bang et al. [54] and Kwon et al. [55] because proteinuria was rarely available for patients in our dataset, and this has likely impaired the estimated performance of these models. Third, we could not reproduce the exact KDIGO definition of CKD because ACR is not routinely collected in UK primary care. Again these limitations are unlikely to influence the implications of our findings for current practice. Finally, we had missing values for ethnicity and considered patients for which there was no ethnicity information recorded as if they were of White British ethnicity. Poor recording of ethnicity is an acknowledged issue in the NHS [68]. However, because of the regional nature of our data, which covers only the city of Salford (England, UK), where white prevalence is higher than 90 % [34], we believe that this did not affect our findings.

## Conclusion

To conclude, we have provided an independent, external validation of CKD prediction models with data that will soon be available in most parts of the UK. All included models had good discriminative performance, but most of them were poorly calibrated. Although no model was ideal, QKidney® [36] performed best, and could support a high-risk approach to CKD prevention in primary care. This study underlines the need for ongoing (re)calibration of clinical prediction models in their contexts of use.

## Abbreviations

ACR, Albumin-creatinine ratio; AUC, area under receiving operating characteristic curve; CKD, Chronic kidney disease; CKD-EPI, Chronic Kidney Disease Epidemiology Collaboration; CPH, Cox proportional hazards; eGFR, estimated Glomerular Filtration Rate; EHR, electronic health record; KDIGO, Kidney Disease: Improving Global Outcomes; MAPE, mean absolute prediction error; MDRD, Modification of Diet in Renal Disease; PPV, positive predictive value; SD, standard deviation; SIR, Salford Integrated Record

## Additional files

Additional file 1: Read codes used to extract the data from the Salford Integrated Record. (CSV 518 kb) Additional file 2: R script to test QKidney®. (R 6 kb) Additional file 3: Full details about included CKD prediction models and sensitivity analyses results. (DOCX 189 kb)

## References

[1] Meguid El Nahas A, Bello AK (2005). Chronic kidney disease: the global challenge. Lancet.

[2] Barsoum RS (2006). Chronic kidney disease in the developing world. N Engl J Med.

[3] Schoolwerth AC, Engelgau MM, Hostetter TH, Rufo KH, Chianchiano D, McClellan WM (2006). Chronic kidney disease: a public health problem that needs a public health action plan. Prev Chronic Dis.

[4] Mills KT, Xu Y, Zhang W, Bundy JD, Chen C-S, Kelly TN (2015). A systematic analysis of worldwide population-based data on the global burden of chronic kidney disease in 2010. Kidney Int.

[5] Coresh J, Selvin E, Stevens LA, Manzi J, Kusek JW, Eggers P (2007). Prevalence of chronic kidney disease in the United States. JAMA.

[6] United States Renal Data System. USRDS 2013 Annual Data Report: Atlas of Chronic Kidney Disease and End-Stage Renal Disease in the United States. Bethesda, MD: National Institutes of Health, National Institute of Diabetes and Digestive and Kidney Diseases; 2013. http://www.usrds.org/atlas.aspx. Accessed April 2016.

[7] Jameson K, Jick S, Hagberg KW, Ambegaonkar B, Giles A, O’Donoghue D (2014). Prevalence and management of chronic kidney disease in primary care patients in the UK. Int J Clin Pract.

[8] Kerr M, Bray B, Medcalf J, O’Donoghue DJ, Matthews B (2012). Estimating the financial cost of chronic kidney disease to the NHS in England. Nephrol Dial Transplant.

[9] Grams ME, Coresh J (2013). Assessing risk in chronic kidney disease: a methodological review. Nat Rev Nephrol.

[10] Radhakrishnan J, Remuzzi G, Saran R, Williams DE, Rios-Burrows N, Powe N (2014). Taming the chronic kidney disease epidemic: a global view of surveillance efforts. Kidney Int.

[11] National Institute for Health and Care Excellence (NICE) UK. Chronic kidney disease: early identification and management of chronic kidney disease in adults in primary and secondary care. 2014. https://www.nice.org.uk/guidance/cg182. Accessed April 2016. 25340245

[12] Glassock RJ, Winearls CG (2008). Routine reporting of estimated glomerular filtration rate: not ready for prime time. Nat Clin Pract Nephrol.

[13] Steyerberg EW (2010). Clinical Prediction Models: A Practical Approach to Development, Validation, and Updating.

[14] Collins GS, Omar O, Shanyinde M, Yu L-M (2013). A systematic review finds prediction models for chronic kidney disease were poorly reported and often developed using inappropriate methods. J Clin Epidemiol.

[15] Echouffo-Tcheugui JB, Kengne AP. Risk models to predict chronic kidney disease and its progression: a systematic review. Remuzzi G, editor. PLoS Med. 2012;9(11):e1001344. 10.1371/journal.pmed.1001344 PMC3502517 23185136

[16] Collins GS, Moons KGM (2012). Comparing risk prediction models. BMJ..

[17] Mogueo A, Echouffo-Tcheugui JB, Matsha TE, Erasmus RT, Kengne AP (2015). Validation of two prediction models of undiagnosed chronic kidney disease in mixed-ancestry South Africans. BMC Nephrol.

[18] Collins GS, Reitsma JB, Altman DG, Moons KGM (2015). Transparent reporting of a multivariable prediction model for individual prognosis or diagnosis (TRIPOD): the TRIPOD Statement. BMC Med..

[19] Moons KGM, Altman DG, Reitsma JB, Ioannidis JPA, Macaskill P, Steyerberg EW (2015). Transparent reporting of a multivariable prediction model for individual prognosis or diagnosis (TRIPOD): explanation and elaboration. Ann Intern Med.

[20] Ando M, Yanagisawa N, Ajisawa A, Tsuchiya K, Nitta K (2011). A simple model for predicting incidence of chronic kidney disease in HIV-infected patients. Clin Exp Nephrol.

[21] Stevens PE, Levin A (2013). Evaluation and management of chronic kidney disease: synopsis of the kidney disease: improving global outcomes 2012 clinical practice guideline. Ann Intern Med.

[22] Levey AS, Bosch JP, Lewis JB, Greene T, Rogers N, Roth D (1999). A more accurate method to estimate glomerular filtration rate from serum creatinine: a new prediction equation. Modification of Diet in Renal Disease Study Group. Ann Intern Med.

[23] Bello A, Thompson S, Lloyd A, Hemmelgarn B, Klarenbach S, Manns B (2012). Multiple versus single and other estimates of baseline proteinuria status as predictors of adverse outcomes in the general population. Am J Kidney Dis.

[24] NHS England. Read Codes. http://www.connectingforhealth.nhs.uk/systemsandservices/data/uktc/readcodes. Accessed 16 June 2014.

[25] Roland M (2004). Linking physicians’ pay to the quality of care--a major experiment in the United Kingdom. N Engl J Med.

[26] Sutton M, Elder R, Guthrie B, Watt G (2010). Record rewards: the effects of targeted quality incentives on the recording of risk factors by primary care providers. Health Econ.

[27] Taggar JS, Coleman T, Lewis S, Szatkowski L (2012). The impact of the Quality and Outcomes Framework (QOF) on the recording of smoking targets in primary care medical records: cross-sectional analyses from The Health Improvement Network (THIN) database. BMC Public Health..

[28] Springate DA, Kontopantelis E, Ashcroft DM, Olier I, Parisi R, Chamapiwa E (2014). ClinicalCodes: an online clinical codes repository to improve the validity and reproducibility of research using electronic medical records. PLoS One.

[29] Doran T, Kontopantelis E, Valderas JM, Campbell S, Roland M, Salisbury C (2011). Effect of financial incentives on incentivised and non-incentivised clinical activities: longitudinal analysis of data from the UK Quality and Outcomes Framework. BMJ..

[30] Kontopantelis E, Springate D, Reeves D, Ashcroft DM, Valderas JM, Doran T (2014). Withdrawing performance indicators: retrospective analysis of general practice performance under UK Quality and Outcomes Framework. BMJ..

[31] Fairhurst C, Watt I, Martin F, Bland M, Brackenbury WJ (2014). Exposure to sodium channel-inhibiting drugs and cancer survival: protocol for a cohort study using the QResearch primary care database. BMJ Open.

[32] Nicholson A, Ford E, Davies KA, Smith HE, Rait G, Tate AR (2013). Optimising use of electronic health records to describe the presentation of rheumatoid arthritis in primary care: a strategy for developing code lists. PLoS One.

[33] Crooks CJ, West J, Card TR (2013). Comorbidities affect risk of nonvariceal upper gastrointestinal bleeding. Gastroenterology.

[34] Salford City Council. BME communities. Salford City Council; 2011. http://ukcensusdata.com/salford-e08000006/ethnic-group-qs201ew#sthash.yRrbrTwP.xjedN1sT.dpbs. Accessed 7 Aug 2016.

[35] Buuren van S, Groothuis-Oudshoorn K. mice: Multivariate Imputation by Chained Equations in R. J Stat Software. 2011. http://doc.utwente.nl/78938/1/Buuren11mice.pdf. Accessed 9 June 2014.

[36] Hippisley-Cox J, Coupland C (2010). Predicting the risk of chronic kidney disease in men and women in England and Wales: prospective derivation and external validation of the QKidney Scores. BMC Fam Pract..

[37] Robin X, Turck N, Hainard A, Tiberti N, Lisacek F, Sanchez J-C (2011). pROC: an open-source package for R and S+ to analyze and compare ROC curves. BMC Bioinformatics.

[38] Newson R (2006). Confidence intervals for rank statistics: Somers’ D and extensions. Stata J.

[39] Jr Harrell FE. Package “Hmisc”. 2014. http://cran.r-project.org/web/packages/Hmisc/Hmisc.pdf. Accessed April 2016.

[40] Verburg IWM, de Keizer NF, de Jonge E, Peek N (2014). Comparison of regression methods for modeling intensive care length of stay. PLoS One.

[41] Cox DR (1958). Two further applications of a model for binary regression. Biometrika.

[42] Kundu S, Aulchenko YS, van Duijn CM, Janssens ACJW (2011). PredictABEL: an R package for the assessment of risk prediction models. Eur J Epidemiol.

[43] Youden WJ (1950). Index for rating diagnostic tests. Cancer.

[44] Debray TPA, Vergouwe Y, Koffijberg H, Nieboer D, Steyerberg EW, Moons KGM (2015). A new framework to enhance the interpretation of external validation studies of clinical prediction models. J Clin Epidemiol.

[45] Vickers AJ, Elkin EB (2006). Decision curve analysis: a novel method for evaluating prediction models. Med Decis Making.

[46] Rose G (2001). Sick individuals and sick populations. Int J Epidemiol.

[47] Moyer VA (2012). Screening for chronic kidney disease: U.S. Preventive Services Task Force recommendation statement. Ann Intern Med.

[48] Levey AS, Stevens LA, Schmid CH, Zhang YL, Castro AF, Feldman HI (2009). A new equation to estimate glomerular filtration rate. Ann Intern Med.

[49] Fox CS, Gona P, Larson MG, Selhub J, Tofler G, Hwang S-J (2010). A multi-marker approach to predict incident CKD and microalbuminuria. J Am Soc Nephrol.

[50] Halbesma N, Jansen DF, Heymans MW, Stolk RP, de Jong PE, Gansevoort RT (2011). Development and validation of a general population renal risk score. Clin J Am Soc Nephrol.

[51] Chien K-L, Lin H-J, Lee B-C, Hsu H-C, Lee Y-T, Chen M-F (2010). A prediction model for the risk of incident chronic kidney disease. Am J Med.

[52] O’Seaghdha CM, Lyass A, Massaro JM, Meigs JB, Coresh J, D’Agostino RB (2012). A risk score for chronic kidney disease in the general population. Am J Med.

[53] Kshirsagar AV, Bang H, Bomback AS, Vupputuri S, Shoham DA, Kern LM (2008). A simple algorithm to predict incident kidney disease. Arch Intern Med.

[54] Bang H, Vupputuri S, Shoham DA, Klemmer PJ, Falk RJ, Mazumdar M (2007). SCreening for Occult REnal Disease (SCORED): a simple prediction model for chronic kidney disease. Arch Intern Med.

[55] Kwon K-S, Bang H, Bomback AS, Koh D-H, Yum J-H, Lee J-H (2012). A simple prediction score for kidney disease in the Korean population. Nephrology (Carlton).

[56] Thakkinstian A, Ingsathit A, Chaiprasert A, Rattanasiri S, Sangthawan P, Gojaseni P (2011). A simplified clinical prediction score of chronic kidney disease: a cross-sectional-survey study. BMC Nephrol.

[57] O’Seaghdha CM, Yang Q, Wu H, Hwang S-J, Fox CS (2012). Performance of a genetic risk score for CKD stage 3 in the general population. Am J Kidney Dis.

[58] Hippisley-Cox J, Coupland C, Brindle P (2014). The performance of seven QPrediction risk scores in an independent external sample of patients from general practice: a validation study. BMJ Open..

[59] Collins G, Altman D (2012). Predicting the risk of chronic kidney disease in the UK: an evaluation of QKidney® scores using a primary care database. Br J Gen Pract.

[60] de Lusignan S, Tomson C, Harris K, van Vlymen J, Gallagher H (2011). Creatinine fluctuation has a greater effect than the formula to estimate glomerular filtration rate on the prevalence of chronic kidney disease. Nephron Clin Pract.

[61] Walsh C, Hripcsak G (2014). The effects of data sources, cohort selection, and outcome definition on a predictive model of risk of thirty-day hospital readmissions. J Biomed Inform..

[62] Levin A, Stevens PE (2014). Summary of KDIGO 2012 CKD Guideline: behind the scenes, need for guidance, and a framework for moving forward. Kidney Int.

[63] Carter JL, Stevens PE, Irving JE, Lamb EJ (2011). Estimating glomerular filtration rate: comparison of the CKD-EPI and MDRD equations in a large UK cohort with particular emphasis on the effect of age. QJM.

[64] Satagopan JM, Ben-Porat L, Berwick M, Robson M, Kutler D, Auerbach AD (2004). A note on competing risks in survival data analysis. Br J Cancer.

[65] Putter H, Fiocco M, Geskus RB (2007). Tutorial in biostatistics: competing risks and multi-state models. Stat Med.

[66] Goldstein BA, Navar AM, Pencina MJ, Ioannidis JPA. Opportunities and challenges in developing risk prediction models with electronic health records data: a systematic review. J Am Med Informatics Assoc. 2016;pii:ocw042. Ahead of print. 10.1093/jamia/ocw042 PMC5201180 27189013

[67] Akbarov A, Williams R, Brown B, Mamas M, Peek N, Buchan I (2015). A two-stage dynamic model to enable updating of clinical risk prediction from longitudinal health record data: illustrated with kidney function. Stud Health Technol Inform..

[68] Hull SA, Mathur R, Badrick E, Robson J, Boomla K (2011). Recording ethnicity in primary care: assessing the methods and impact. Br J Gen Pract.

