# Supplementary material for: An external validation of models to predict the onset of chronic kidney disease using population-based electronic health records from Salford, UK
Source: BMC Med. 2016 Jul 12;14:104. doi: 10.1186/s12916-016-0650-2 (PMC4940699; doi:10.1186/s12916-016-0650-2)
Supplement: Additional file 3: — Full details about included CKD prediction models and sensitivity analyses results. (DOCX 189 kb) [file 12916_2016_650_MOESM3_ESM.docx]

**Supplementary material**

**Supplementary material 1** Mean absolute prediction error (MAPE) definition**.**

$$\boldsymbol{MAPE=}\frac{\boldsymbol{1}}{\boldsymbol{n}}\sum_{\boldsymbol{i=1}}^{\boldsymbol{n}} \left| \hat{\boldsymbol{y}_{\boldsymbol{i}}}\boldsymbol{-}\boldsymbol{y}_{\boldsymbol{i}} \right|$$

Where n is the total number of patients $\hat{\boldsymbol{y}_{\boldsymbol{i}}}$ is the predicted risk of CKD onset for the ith patient and $\boldsymbol{y}_{\boldsymbol{i}}$ is the observed outcome (CKD onset or not) for the same patient.

**Table S1** Population characteristics in included models’ original studies.

| **Parameter** | **Bang *et al.*** [51] | **Chien *et al*** [48] | | | **Kshirsagar *et. al.*** [50] | | **Kwon *et al.*** [52] | **O'seaghdha *et al.*** [49] | **Thakkinstian  *et al*** [53] | **QKidney** [35] |
| --- | --- | --- | --- | --- | --- | --- | --- | --- | --- | --- |
|  | **Total** | **Total** | **No CKD** | **CKD** | **No CKD** | **CKD** | **Total** | **Total** | **Total** | **Total** |
| Number of patients | 8,530 | 4,978 | 5,168 | 190 | 9,470 | 1,605 | 6,565 | 2,490 | 3,459 | 1,574,749 |
| Number of cases (%) | 601 (7.5) | 190 (3.7) | / | / | / | / | 100 (1.5) | 229 (9.2) | 606 (17.5) | 23,786 (1.5) |
| Age (mean, SD) | 46 (31.4) | 51.2 (10.5) | 50.8 (10.3) | 60.9 (9.4) | 57 (9) | 62 (9) | 44.2 (0.4) | 57.1 (8.9) | 45.2 (0.79) | 47.3 (11.1) |
| Gender - Female (%) | 52% | 36.7% | 36.9% | 32.1% | 56% | 59% | 50.0% | 53.0% | 54.5% | 49.22% |
| Townsend score (mean, SD) |  |  |  |  |  |  |  |  |  | -0.5 (3.4) |
| Ethnicity | White 72%  Black 10%  Hispanic 14% | Chinese 100% | Chinese 100% | Chinese 100% | White 78% | White 83% | Asian 100% | White 100% | Asian 100% | White 95% |
| Smoking status |  |  |  |  |  |  |  |  |  | Non-smoker 51.11%  Ex-smoker 18.21  Light smoker 6.43%  Moderate smoker 7.89%  Heavy smoker 6.41% |
| BMI [kg/m^2] (mean, SD) | 28.0 (12.9) | 23.9 (3.1) | 23.9 (3.1) | 24.7 (3.0) |  |  | 23.6 (0.1) |  | 24 (0.2) | 26.7 (4.7) |
| Diastolic blood pressure [mmHg] (mean, SD) |  | 73.7(10.5) | 73.5(10.5) | 77.6 (11.0) | 73 (11) | 73 (12) |  |  |  |  |
| Systolic blood pressure [mmHg] (mean, SD) |  | 123.2 (15.8) | 122.9 (15.7) | 131.3 (16.2) | 122 (19) | 132 (22) |  | 126 (18) |  | 133.1 (19.5) |
| eGFR [mL/min/1.73 m^2^]  (mean, SD) | 94 (48.9) | 82.2 (13.1) | 82.7(13.0) | 68.3(7.0) |  |  | 85.9 (0.7) | 92 (23) |  |  |
| Anemia (%) | 2.7% | 2.9% | 2.9% | 1.6% | 2% | 3% | 8.1% | 7.4% |  |  |
| Postpondrial glucose [mg/dl]  (mean +/- std) |  | 118.4(52.5) | 118.0 (51.2) | 130.9 (77.7) |  |  |  |  |  |  |
| HbA1c [%](mean, SD) |  | 5.52 (0.71) | 5.51(0.70) | 5.78 (0.98) |  |  |  |  |  |  |
| Uric acid [mg/dl] (mean, SD) |  | 6.11(1.46) | 6.09 (1.46) | 6.55 (1.47) |  |  |  |  |  |  |
| CRP [mg/dl] (mean, SD) |  | 0.15 (0.37) | 0.15 (0.35) | 0.23 (0.69) |  |  |  |  |  |  |
| Anemia (%) | 2.7% | 2.9% | 2.9% | 1.6% | 2% | 3% | 8.1% | 7.4% |  |  |
| Proteinuria (%) | 10% |  |  |  |  |  | 10.3% | 17.1% |  |  |
| Quantitative albuminuria (%) |  |  |  |  |  |  |  | 9.2% |  |  |
| High-density lipoprotein cholesterol level [mg/dl] (mean, SD) | 51.3 (34.2) |  |  |  |  |  |  |  |  |  |
| Hypertension (%) | 34% | 23.9% | 23.0% | 47.9% | 36% | 55% | 22.5% | 35.3% | 27.5% |  |
| Hypertensive treatment (%) |  |  |  |  |  |  |  |  |  | 9.94% |
| Diabetes Mellitus (%) | 8% | Type 2 12.2% | Type 2 11.8% | Type 2 23.7% | 9% | 17% | 8.3% |  | 11.9% | Type 1 0.28%  Type 2 3.12% |
| History of cardiovascular disease (%) |  |  |  |  | 8% | 17% | 3.2% | 8.3% |  | 4.5% |
| History of heart failure (%) |  |  |  |  | 0.7 | 2.3 |  |  |  | 0.5% |
| History of stroke (%) |  | 0.4% | 0.3% | 2.1% |  |  |  |  |  |  |
| Peripheral vascular disease (%) | 2.7% |  |  |  | 4% | 9% |  |  |  | 1% |
| Kidney stones (%) |  |  |  |  |  |  |  |  | 5.0% | 0.68% |
| Rheumatoid arthritis (%) |  |  |  |  |  |  |  |  |  | 0.76% |
| Systemic lupus erythematosus (%) |  |  |  |  |  |  |  |  |  | 0.07% |
| NSAIDs use (%) |  |  |  |  |  |  |  |  |  | 27.04% |
| Family history of kidney disease (%) |  |  |  |  |  |  |  |  |  | 0.05 |

**Table S2** Coefficients of validated models. QKidney included also Townsend score, fractional polynomial terms for age, body mass index and systolic blood pressure as well as interactions between the age terms and type 1 diabetes, type 2 diabetes and treated hypertension (full details in supplementary file 1). *Cox proportional hazard model (baseline hazard=0.9632).**Intercept estimated from summary measures and CKD prevalence from development dataset.

| **Parameter** | **Bang *et al.*** [51] | **Chien *et al**** [48] | **Kshirsagar *et. al.*** [50] | **Kwon *et al.*** [52] | **O'seaghdha *et al.*** [49] | **Thakkinstian  *et al*** [53] | **QKidney®** [35] | | |
| --- | --- | --- | --- | --- | --- | --- | --- | --- | --- |
| Intercept | -5.4 | -6.8 | -4.1** | -4.4** | -8.3** | -2.8 | **F** | **M** | **Strata** |
| Age | 1.55 [50-59]  2.31 [60-69]  3.23 [>=70] | 0.08 [per year] | 0.63 [50-59]  1.33 [60-69]  1.46 [>70] | 1.16 [50-59]  1.91 [60-69]  2.71 [>70] | 0.05  [per year] | 0.6 [40-59]  1.4 [60-69]  2.1 [>70] | Included | Included |  |
| Gender - Female | 0.29 |  | 0.13 | 0.4 |  |  |  |  |  |
| Anaemia | 0.93 |  | 0.48 | 0.94 |  |  |  |  |  |
| Hypertension (yes/no) | 0.45 |  | 0.55 | 0.48 | 0.32 | 0.8 |  |  |  |
| Type 1 Diabetes Mellitus (yes/no) | 0.44 |  | 0.33 | 0.73 |  | 0.9 | 2.10 | 2.51 |  |
| Type 2 Diabetes Mellitus (yes/no) | 0.44 | 0.37 | 0.33 | 0.73 | 0.29 | 0.9 | 1.50 | 1.80 |  |
| History of cardiovascular disease (yes/no) | 0.59 |  | 0.26 | 0.60 |  |  | 0.32 | 0.33 |  |
| History of heart failure (yes/no) | 0.45 |  | 0.50 |  |  |  | 0.82 | 1.04 |  |
| Hystory of stroke (yes/no) |  | 1.24 |  |  |  |  |  |  |  |
| Peripheral vascular disease (yes/no) | 0.74 |  | 0.41 |  |  |  | 0.30 | 0.38 |  |
| Proteinuria | 0.83 |  |  | 0.48 |  |  |  |  |  |
| BMI |  | 0.06  [per kg/m^2^] |  |  |  |  | Included | Included |  |
| Diastolic blood pressure |  | 0.02  [per mmHg] |  |  |  |  |  |  |  |
| Kidney stones |  |  |  |  |  | 1 |  |  |  |
| Ethnicity |  |  |  |  |  |  | 0.30  0.44  0.40  0.15  -0.73  -0.57  0.12  0.20 | 0.15  0.69  0.30  0.36  -0.19  0.16  0.31  0.29 | [Indian]  [Pakistani]  [Bangladeshi]  [Other Asian]  [Black Caribbean]  [Black African]  [Chinese]  [Other ethnic group] |
| Treated hypertension (yes/no) |  |  |  |  |  |  | 0.91 | 1.02 |  |
| NSAID use (yes/no) |  |  |  |  |  |  | 0.26 | 0.25 |  |
| Family history of kidney disease (yes/no) |  |  |  |  |  |  | 0.75 | 1.27 |  |
| Rheumatoid arthritis (yes/no) |  |  |  |  |  |  | 0.48 |  |  |
| Systemic lupus erythematosus (yes/no) |  |  |  |  |  |  | 0.88 |  |  |
| Smoking |  |  |  |  |  |  | 0.17  0.27  0.24  0.36 | 0.12  0.14  0.21  0.22 | [Ex-smoker]  [Light smoker]  [Moderate smoker]  [Heavy smoker] |
| Systolic blood pressure |  |  |  |  |  |  | Included | Included |  |
| Townsend score |  |  |  |  |  |  | Included | Included |  |

**Table S3** Weights of validated simplified scoring systems.

| **Parameter** | **Bang *et al.*** [51] | **Chien *et al*** [48] | **Kshirsagar *et. al.*** [50] | **Kwon *et al.*** [52] | **Thakkinstian  *et al*** [53] |
| --- | --- | --- | --- | --- | --- |
| Age | 2 [50-59]  3 [60-69]  4 [>=70] | 3 [45-54]  5 [55-64]  8 [>=65] | 1 [50-59]  2 [60-69]  3 [>=70] | 2 [50-59]  3 [60-69]  4 [>=70] | 1 [<40]  2 [40-59]  4 [60-69]  8 [>=70] |
| Gender - Female | 1 |  | 1 | 1 |  |
| Anaemia | 1 |  | 1 | 1 |  |
| Hypertension (yes/no) | 1 |  | 1 | 1 | 1 [No]  2 [Yes] |
| Type 1 Diabetes Mellitus (yes/no) | 1 |  | 1 | 1 | 1 [No]  3 [Yes] |
| Type 2 Diabetes Mellitus (yes/no) | 1 | 1 | 1 | 1 | 1 [No]  3 [Yes] |
| History of cardiovascular disease (yes/no) | 1 |  | 1 | 1 |  |
| History of heart failure (yes/no) | 1 |  | 1 |  |  |
| Hystory of stroke (yes/no) |  | 4 |  |  |  |
| Peripheral vascular disease (yes/no) | 1 |  | 1 |  |  |
| Proteinuria | 1 |  |  | 1 |  |
| BMI |  | 1 [21-25]  2 [>=26] |  |  |  |
| Diastolic blood pressure |  | 1 [66-79]  2 [>=80] |  |  |  |
| Kidney stones |  |  |  |  | 1 [No]  3 [Yes] |

**Table S4** Discrimination, MAPE and calibration slopes of included models in patients with established risk factors for CKD at inclusion, computed in patients with complete follow-up data (all models and risk scores) and in the full validation cohort (Cox proportional hazards regression models only).

|  | **Study** | **Patients with complete follow-up**  **(N=** **44,183)** | | | **Full validation cohort**  **(N=** **49,002)** | |
| --- | --- | --- | --- | --- | --- | --- |
|  |  | **AUC [CI]** | **MAPE (SD) ^a)^** | **Calibration slope [CI]** | **c-index [CI]** | **MAPE (SD) ^a)^** |
| MODELS | Bang *et al.* [51] | 0.795 [0.798,0.802] | 0.166 (0.24) | 0.93 [0.91,0.95] | NA | NA |
|  | Chien *et al* [48]^b)^ | 0.781 [0.775,0.787] | 0.196 (0.232) | 0.64 [0.62,0.66] | 0.755[0.749,0.761] | 0.166 (0.24) |
|  | QKidney® [35]^b)^ | 0.801 [0.795,0.808] | 0.144 (0.255) | 0.93 [0.91,0.95] | 0.775[0.769,0.781] | 0.196 (0.232) |
|  | Kshirsagar *et. al.* [50] | 0.779 [0.773,0.786] | 0.162 (0.252) | 1.81 [1.77,1.86] | NA | NA |
|  | Kwon *et al.* [52] | 0.794 [0.788,0.800] | 0.212 (0.211) | 0.69 [0.67,0.71] | NA | NA |
|  | O'seaghdha *et al.* [49] | 0.796 [0.790,0.803] | 0.207 (0.221) | 0.54 [0.53,0.56] | NA | NA |
|  | Thakkinstian *et al* [53] | 0.756 [0.749,0.762] | 0.23 (0.227) | 0.49 [0.47,0.50] | NA | NA |
| SIMPLIFIED SCORES | Bang *et al.* [51] | 0.786 [0.780,0.792] | NA | NA | NA | NA |
|  | Chien *et al -* [48] | 0.743 [0.736,0.749] | NA | NA | NA | NA |
|  | Kshirsagar et. al. [50] | 0.785 [0.779,0.790] | NA | NA | NA | NA |
|  | Kwon *et al.* [52] | 0.784 [0.778,0.790] | NA | NA | NA | NA |
|  | Thakkinstian *et al* [53] | 0.763 [0.756,0.770] | NA | NA | NA | NA |

Abbreviations: AUC, area under receiver operating characteristic curve; eGFR, estimated glomerular filtration rate; NA, not applicable; SD, standard deviation; CI, 95% confidence interval.

1. Calculated as mean difference between observed and predicted CKD cases
2. Cox proportional hazard regression model
3. Difference in lower

**Table S5** Models and scores’ AUC and c-index discrimination as well as MAPE by adopting the CKD definition of eGFR<60 ml/min/1.73 m^2^ on a single occasion as well as a CKD 3-5 diagnostic code.

|  | **Study** | **Patients with complete follow-up**  **(N=** **157,096)** | | | **Patients with incomplete follow-up**  **(N=** **170,410)** | |
| --- | --- | --- | --- | --- | --- | --- |
|  |  | **AUC [CI]** | **MAPE (SD) ^a)^** | **Calibration slope [CI]** | **c-index [CI]** | **MAPE (SD) ^a)^** |
| MODELS | Bang *et al.* [51] | 0.839 [0.834,0.844] | 0.076 (0.203) | 1.24 [1.22,1.25] | NA | NA |
|  | Chien *et al* [48]^b)^ | 0.851 [0.847,0.855] | 0.092 (0.197) | 0.82 [0.81,0.83] | 0.841[0.837,0.845] | 0.091 (0.194) |
|  | QKidney® [35]^b)^ | 0.862 [0.858,0.866] | 0.065 (0.211) | 1.29 [1.27,1.30] | 0.852[0.848,0.856] | 0.064 (0.205) |
|  | Kshirsagar *et. al.* [50] | 0.839 [0.835,0.843] | 0.083 (0.205) | 2.24 [2.21,2.27] | NA | NA |
|  | Kwon *et al.* [52] | 0.838 [0.833,0.843] | 0.096 (0.193) | 0.87 [0.86,0.88] | NA | NA |
|  | O'seaghdha *et al.* [49] | 0.859 [0.855,0.864] | 0.096 (0.196) | 0.66 [0.66,0.67] | NA | NA |
|  | Thakkinstian *et al* [53] | 0.845 [0.841,0.849] | 0.18 (0.173) | 0.57 [0.57,0.58] | NA | NA |
| SIMPLIFIED SCORES | Bang *et al.* [51] | 0.836 [0.831,0.841] | NA | NA | NA | NA |
|  | Chien *et al -* [48] | 0.836 [0.831,0.841] | NA | NA | NA | NA |
|  | Kshirsagar et. al. [50] | 0.829 [0.824,0.834] | NA | NA | NA | NA |
|  | Kwon *et al.* [52] | 0.836 [0.831,0.841] | NA | NA | NA | NA |
|  | Thakkinstian *et al* [53] | 0.811 [0.806,0.816] | NA | NA | NA | NA |

Abbreviations: AUC, area under receiver operating characteristic curve; eGFR, estimated glomerular filtration rate; NA, not applicable; SD, standard deviation; CI, 95% confidence interval.

1. Calculated as mean difference between observed and predicted CKD cases
2. Cox proportional hazard regression model

**Table S6** Models and scores’ AUC and c-index discrimination as well as MAPE for the sensitivity analysis by considering patients who died as if they developed CKD.

|  | **Study** | **Patients with complete follow-up**  **(N=169,548)** | | | **Patients with incomplete follow-up**  **(N=179,072)** | |
| --- | --- | --- | --- | --- | --- | --- |
|  |  | **AUC [CI]** | **MAPE (SD) ^a)^** | **Calibration slope [CI]** | **c-index [CI]** | **MAPE (SD) ^a)^** |
| MODELS | Bang *et al.* [51] | 0.886 [0.883,0.889] | -0.028 (0.239) | 1.39 [1.38,1.41] | NA | NA |
|  | Chien *et al* [48]^b)^ | 0.888 [0.885,0.891] | -0.007 (0.238) | 1.05 [1.04,1.06] | 0.875[0.873,0.878] | -0.005 (0.232) |
|  | QKidney® [35]^b)^ | 0.899 [0.896,0.902] | -0.048 (0.24) | 1.48 [1.47,1.49] | 0.886[0.884,0.8819] | -0.045 (0.234) |
|  | Kshirsagar *et. al.* [50] | 0.881 [0.878,0.884] | -0.035 (0.253) | 2.72 [2.70,2.74] | NA | NA |
|  | Kwon *et al.* [52] | 0.881 [0.878,0.884] | 0.007 (0.237) | 1.02 [1.01,1.03] | NA | NA |
|  | O'seaghdha *et al.* [49] | 0.900 [0.897,0.903] | 0.013 (0.232) | 0.87 [0.86,0.88] | NA | NA |
|  | Thakkinstian *et al* [53] | 0.876 [0.873,0.879] | 0.106 (0.245) | 0.76 [0.75,0.77] | NA | NA |
| SIMPLIFIED SCORES | Bang *et al.* [51] | 0.877 [0.874,0.880] | NA | NA | NA | NA |
|  | Chien *et al -* [48] | 0.866 [0.863,0.869] | NA | NA | NA | NA |
|  | Kshirsagar et. al. [50] | 0.868 [0.865,0.871] | NA | NA | NA | NA |
|  | Kwon *et al.* [52] | 0.876 [0.873,0.879] | NA | NA | NA | NA |
|  | Thakkinstian *et al* [53] | 0.868 [0.864,0.872] | NA | NA | NA | NA |

Abbreviations: AUC, area under receiver operating characteristic curve; eGFR, estimated glomerular filtration rate; NA, not applicable; SD, standard deviation; CI, 95% confidence interval.

1. Calculated as mean difference between observed and predicted CKD cases
2. Cox proportional hazard regression model

**Table S7** Models and scores’ AUC and c-index discrimination as well as MAPE by focusing only on patients without missing values in any predictor (complete cases).

|  | **Study** | **Patients with complete follow-up**  **(N=36,092)** | | | **Patients with incomplete follow-up**  **(N=39,283)** | |
| --- | --- | --- | --- | --- | --- | --- |
|  |  | **AUC [CI]** | **MAPE (SD) ^a)^** | **Calibration slope [CI]** | **c-index [CI]** | **MAPE (SD) ^a)^** |
| MODELS | Bang *et al.* [51] | 0.840 [0.834,0.846] | 0.13 (0.223) | 0.95 [0.925,0.97] | NA | NA |
|  | Chien *et al* [48]^b)^ | 0.828 [0.822,0.834] | 0.158 (0.219) | 0.65 [0.632,0.67] | 0.804[0.798,0.81] | 0.168 (0.222) |
|  | QKidney® [35]^b)^ | 0.846 [0.840,0.852] | 0.113 (0.234) | 0.93 [0.908,0.95] | 0.821[0.815,0.827] | 0.12 (0.233) |
|  | Kshirsagar *et. al.* [50] | 0.831 [0.825,0.837] | 0.128 (0.232) | 1.82 [1.773,1.87] | NA | NA |
|  | Kwon *et al.* [52] | 0.839 [0.833,0.845] | 0.164 (0.21) | 0.7 [0.685,0.72] | NA | NA |
|  | O'seaghdha *et al.* [49] | 0.840 [0.834,0.846] | 0.18 (0.222) | 0.54 [0.526,0.55] | NA | NA |
|  | Thakkinstian *et al* [53] | 0.816 [0.809,0.823] | 0.278 (0.202) | 0.47 [0.461,0.49] | NA | NA |
| SIMPLIFIED SCORES | Bang *et al.* [51] | 0.895 [0.891,0.899] | NA | NA | NA | NA |
|  | Chien *et al -* [48] | 0.880 [0.876,0.884] | NA | NA | NA | NA |
|  | Kshirsagar et. al. [50] | 0.891 [0.887,0.895] | NA | NA | NA | NA |
|  | Kwon *et al.* [52] | 0.895 [0.891,0.899] | NA | NA | NA | NA |
|  | Thakkinstian *et al* [53] | 0.869 [0.864,0.874] | NA | NA | NA | NA |

Abbreviations: AUC, area under receiver operating characteristic curve; eGFR, estimated glomerular filtration rate; NA, not applicable; SD, standard deviation; CI, 95% confidence interval.

1. Calculated as mean difference between observed and predicted CKD cases
2. Cox proportional hazard regression model

**Table S8** Models and scores’ AUC and c-index discrimination as well as MAPE by calculating eGFR with the CKD-EPI formula.

|  | **Study** | **Patients with complete follow-up**  **(N=161,949)** | | | **Patients with incomplete follow-up**  **(N=177,102)** | |
| --- | --- | --- | --- | --- | --- | --- |
|  |  | **AUC [CI]** | **MAPE (SD) ^a)^** | **Calibration slope [CI]** | **c-index [CI]** | **MAPE (SD) ^a)^** |
| MODELS | Bang *et al.* [51] | 0.908 [0.905,0.911] | 0.069 (0.17) | 1.05 [1.04,1.06] | NA | NA |
|  | Chien *et al* [48]^b)^ | 0.910 [0.907,0.913] | 0.082 (0.168) | 0.8 [0.79,0.8] | 0.9[0.897,0.903] | 0.085 (0.169) |
|  | QKidney® [35]^b)^ | 0.920 [0.918,0.922] | 0.053 (0.176) | 1.26 [1.25,1.27] | 0.91[0.907,0.913] | 0.054 (0.173) |
|  | Kshirsagar *et. al.* [50] | 0.905 [0.902,0.908] | 0.074 (0.174) | 1.96 [1.94,1.98] | NA | NA |
|  | Kwon *et al.* [52] | 0.905 [0.902,0.908] | 0.093 (0.165) | 0.74 [0.74,0.75] | NA | NA |
|  | O'seaghdha *et al.* [49] | 0.920 [0.917,0.923] | 0.088 (0.169) | 0.66 [0.65,0.66] | NA | NA |
|  | Thakkinstian *et al* [53] | 0.902 [0.899,0.905] | 0.177 (0.159) | 0.54 [0.53,0.54] | NA | NA |
| SIMPLIFIED SCORES | Bang *et al.* [51] | 0.895 [0.891,0.899] | NA | NA | NA | NA |
|  | Chien *et al -* [48] | 0.880 [0.876,0.884] | NA | NA | NA | NA |
|  | Kshirsagar et. al. [50] | 0.891 [0.887,0.895] | NA | NA | NA | NA |
|  | Kwon *et al.* [52] | 0.895 [0.891,0.899] | NA | NA | NA | NA |
|  | Thakkinstian *et al* [53] | 0.869 [0.864,0.874] | NA | NA | NA | NA |

Abbreviations: AUC, area under receiver operating characteristic curve; eGFR, estimated glomerular filtration rate; NA, not applicable; SD, standard deviation; CI, 95% confidence interval.

1. Calculated as mean difference between observed and predicted CKD cases
2. Cox proportional hazard regression model

**Table S9** Models and scores’ AUC and c-index discrimination as well as MAPE by using a time-horizon of 4-year.

|  | **Study** | **Patients with complete follow-up**  **(N=172,984)** | | | **Patients with incomplete follow-up**  **(N=178,399)** | |
| --- | --- | --- | --- | --- | --- | --- |
|  |  | **AUC [CI]** | **MAPE (SD) ^a)^** | **Calibration slope [CI]** | **c-index [CI]** | **MAPE (SD) ^a)^** |
| MODELS | Bang *et al.* [51] | 0.902 [0.897,0.907] | 0.059 (0.143) | 0.67 [0.66,0.68] | NA | NA |
|  | Chien *et al* [48]^b)^ | 0.901 [0.898,0.904] | 0.075 (0.149) | 0.49 [0.48,0.49] | 0.891[0.888,0.895] | 0.081 (0.155) |
|  | QKidney® [35]^b)^ | 0.914 [0.91,0.917] | 0.038 (0.146) | 1 [0.99,1.02] | 0.904[0.9,0.907] | 0.041 (0.148) |
|  | Kshirsagar *et. al.* [50] | 0.9 [0.896,0.904] | 0.062 (0.14) | 1.26 [1.25,1.28] | NA | NA |
|  | Kwon *et al.* [52] | 0.9 [0.896,0.904] | 0.087 (0.148) | 0.47 [0.47,0.48] | NA | NA |
|  | O'seaghdha *et al.* [49] | 0.911 [0.908,0.914] | 0.085 (0.161) | 0.4 [0.39,0.41] | NA | NA |
|  | Thakkinstian *et al* [53] | 0.896 [0.892,0.890] | 0.176 (0.158) | 0.33 [0.33,0.34] | NA | NA |
| SIMPLIFIED SCORES | Bang *et al.* [51] | 0.895 [0.891,0.899] | NA | NA | NA | NA |
|  | Chien *et al -* [48] | 0.88 [0.876,0.884] | NA | NA | NA | NA |
|  | Kshirsagar et. al. [50] | 0.891 [0.887,0.895] | NA | NA | NA | NA |
|  | Kwon *et al.* [52] | 0.895 [0.891,0.899] | NA | NA | NA | NA |
|  | Thakkinstian *et al* [53] | 0.869 [0.864,0.874] | NA | NA | NA | NA |

Abbreviations: AUC, area under receiver operating characteristic curve; eGFR, estimated glomerular filtration rate; NA, not applicable; SD, standard deviation; CI, 95% confidence interval.

1. Calculated as mean difference between observed and predicted CKD cases
2. Cox proportional hazard regression model
